# A Cross-Sectional Observational Study to Assess the Efficacy of Triglyceride to High-Density Lipoprotein Ratio as a Marker of Insulin Resistance in Subjects of Central Rural India

**DOI:** 10.7759/cureus.58612

**Published:** 2024-04-19

**Authors:** Khalid Khan, Sabiha Quazi, Nandkishor J Bankar, Anil Wanjari, Rajesh Gosavi, Prashant Joshi, Sunil Gupta

**Affiliations:** 1 Medicine, Datta Meghe Medical College, Datta Meghe Institute of Higher Education and Research (Deemed to be University), Nagpur, IND; 2 Dermatology, Datta Meghe Medical College, Datta Meghe Institute of Higher Education and Research (Deemed to be University), Nagpur, IND; 3 Microbiology, Jawaharlal Nehru Medical College, Datta Meghe Institute of Medical Science (Deemed to be University), Wardha, IND; 4 Medicine, Jawaharlal Nehru Medical College, Datta Meghe Institute of Medical Science (Deemed to be University), Wardha, IND; 5 Medicine, All India Institute of Medical Sciences, Nagpur, IND; 6 Diabetology, Sunil's Diabetes Care n' Research Centre, Nagpur, IND

**Keywords:** triglyceride to high density lipoprotein ratio, obesity, one health, rural health, metabolic obesity, surrogate marker, central india, insulin resistance, tg/hdl ratio

## Abstract

Introduction: The rising prevalence of insulin resistance (IR), obesity, and its complications in India is due to lifestyle changes, eating patterns, stress, and genetic factors. Markers for IR are often expensive, invasive, or impractical for use in economically disadvantaged or remote areas. To address this, we evaluated the efficacy of the triglyceride to high-density lipoprotein (TG/HDL) ratio as a simple, reliable, accessible, and affordable surrogate marker of IR in comparison to the homeostatic model assessment for insulin resistance (HOMA-IR).

Methods: This cross-sectional observational study was performed at a tertiary care center in central India and included 815 subjects aged 18 to 60 years after excluding those with systemic diseases, drugs affecting weight, or pregnant or lactating women. Descriptive and inferential statistical analysis was done to represent the study findings.

Results: Males and obese subjects were more insulin resistant than females and non-obese subjects, respectively. The TG/HDL had a sensitivity of 91.81%, a specificity of 92.88%, a positive predictive value of 94.46%, and a negative predictive value of 89.56%, with a diagnostic accuracy of 92.27% when compared to HOMA-IR.

Conclusion: We concluded that TG/HDL serves as a simple, affordable, and accurate marker of IR in a diverse population of central India. There is a definite scope to use the same for large-scale screening, epidemiological research, and routine clinical practice.

## Introduction

Obesity is a complex multifactorial disease that results from the disproportionate accumulation of excess body fat, which leads to negative effects on health [1]. Anthropometric derangement is the consequence of metabolically disturbed health [2]. Obesity is measured by various parameters; the most commonly used one is body mass index (BMI). According to this, people from the Asia Pacific region with a BMI of ≥25 kg/m^2^ are said to be obese, while for the rest of the world, the cut-off value as per the World Health Organization (WHO) is ≥30 kg/m^2^ [3]. Metabolic consequences of obesity are prediabetes, type 2 diabetes mellitus (T2DM), hypertension (HTN), angina, cardiovascular diseases (CVD), arthritis, sleep disorders, and dyslipidemias [4]. The ultimate reason behind these derangements is insulin resistance (IR), a physiological condition in which cells are unable to react to the hormone insulin as they normally should [5]. For these disorders to be prevented and early intervention to be successful, IR detection at an early stage is essential. However, many clinical settings, particularly those with limited resources, find it very difficult, costly, and impractical to use classic or gold-standard methods of evaluating insulin sensitivity, like the hyperinsulinemic-euglycemic clamp technique. Its use is not only questionable in day-to-day practice but also in large experimental studies due to various practical reasons, like cost, time consumption, and invasiveness [6].

Obesity in India is a rising concern for physicians as well as the general population of India. Its rising prevalence, especially in the last 50 years [2], is the result of a transition in lifestyle and dietary habits [1]. Insulin resistance can exist in anthropometrically non-obese individuals. The prevalence of IR in non-obese Indians varies from 11% to 43% [7], depending on the geographical location, urbanization, economic strata [7, 8], and methods and tests used to calculate it. There are several direct or indirect markers of IR, like the euglycemic-hyperinsulinemic clamp test, the homeostasis model assessment for IR (HOMA-IR), the quantitative insulin-sensitivity check index (QUICKI), oral glucose tolerance tests (OGTT), and various inflammatory markers like adiponectin, C-reactive protein (CRP), and interleukin 1 receptor antagonist (IL-1RA) [9-11]. These current indicators of IR are often complex, invasive, expensive, or not suitable for everyday clinical use, especially in remote areas or widespread epidemiological studies [12]. As a result, there's a pressing need for simpler, more cost-effective, feasible, and trustworthy alternative measures of insulin resistance that are easily applicable in both medical practice and research contexts.

The triglyceride to high-density lipoprotein ratio (TG/HDL) has been suggested and accepted as an alternative indicator of IR [12]. This ratio gained popularity and uniqueness as it indicated the impact of IR on both lipolysis and lipoprotein metabolism in the body [13]. Research has demonstrated the effectiveness of the TG/HDL ratio as an IR marker in children and adolescents in and around India [14, 15]. However, there is a lack of such studies among adults in central India. Therefore, this study was conducted with the objective of evaluating the TG/HDL ratio's efficacy against HOMA-IR in central rural India, encompassing both obese and non-obese individuals, to assess the utility of this ratio in a demographically unique, diverse, genetically susceptible, and economically poor population so that a simple, affordable, non-invasive, and readily accessible potential marker of IR can be found.

## Materials and methods

The present cross-sectional study was conducted in the Department of Medicine of Acharya Vinoba Bhave Rural Hospital (AVBRH), a tertiary care hospital attached to the Jawaharlal Nehru Medical College (JNMC), Wardha in central India. The study included 815 subjects, selected on a unit working day (once a week, convenience sampling method), between the ages of 18 and 60, for a duration of three years (September 2019 to August 2022), who were visiting the Department of Medicine and were willing to participate in the study. Subjects who were diagnosed as diabetic or hypertensive for the first time at the time of inclusion or had other habits were included in the study. Pregnant or lactating women, or subjects consuming medications like hormonal pills, lipid-lowering agents, on weight reduction treatment or chemotherapy, or suffering from diseases causing fluid retention, weight gain, or weight loss like chronic kidney disease (CKD), cirrhosis of the liver with portal hypertension with ascites, cancer, hypothyroidism, hyperthyroidism, and Cushing syndrome, or not willing to participate, non-cooperative, or non-consenting were excluded.

**Figure 1:**
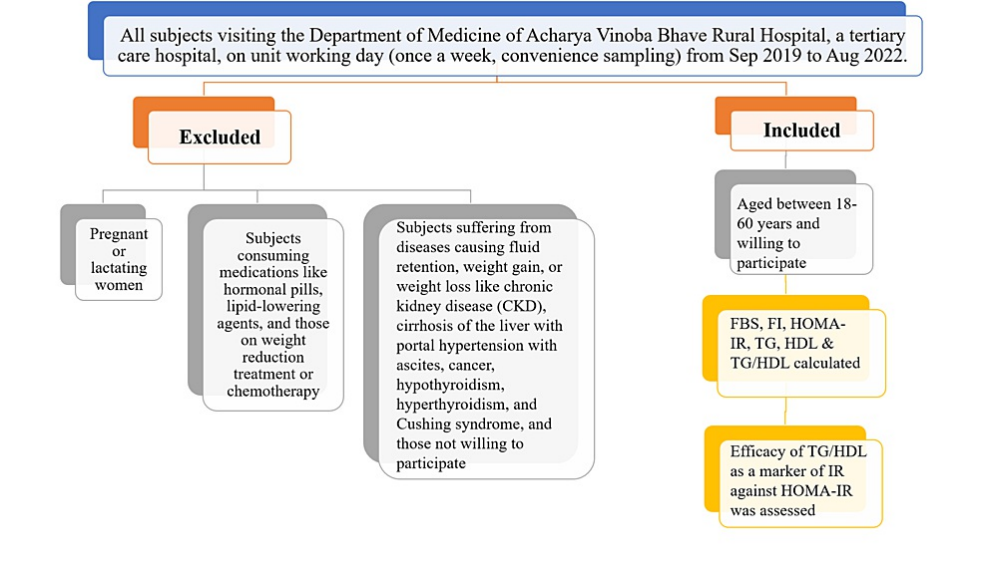
Flowchart of the study methodology FBS: fasting blood sugar (mg/dL); FI: fasting insulin (μIU/ml); HOMA-IR: homeostatic model assessment of insulin resistance; TG: triglycerides (mg/dL); HDL: high-density lipoprotein (mg/dL); TG/HDL: triglyceride to high-density lipoprotein ratio; IR: insulin resistance

After getting ethical approval, informed consent in written form was obtained from the subjects. Enrolled subjects’ personal data and data related to our study objectives were recorded in a proforma. Blood was withdrawn with all possible aseptic techniques from the median cubital vein and spun in a centrifugation machine to separate serum. The serum was carefully extracted and processed in a biochemical analyzer machine to obtain the fasting blood sugar (FBS in mg/dl), insulin levels (fasting insulin (FI) in µIU/ml), and lipid profile (mg/dl). The HOMA-IR was calculated by using the formula FBS x FI/405, and TG was divided by HDL values to get the TG/HDL ratio [11, 14]. All the variables, like FBS, FI, HOMA-IR, TG, HDL, and TG/HDL, were recorded in the proforma. Statistical analysis was done using descriptive statistics, namely mean, median, standard deviation, minimum, and maximum, and inferential statistics, namely the T-test, chi-square test, sensitivity, specificity, and receiver operating characteristic curve (ROC), using SPSS Statistics for Windows, Version 17.0 (SPSS Inc., Chicago, IL) and Prism version 6.0 (GraphPad Software, La Jolla, CA). A p-value <0.05 was considered statistically significant. A flowchart of the study methodology is presented in Figure 1

We compared the efficacy of TG/HDL as a marker of IR by comparing it with HOMA-IR. We assessed the efficacy under the following headings: (1) sensitivity (true positive rate), which is the probability that a test will be positive when the disease is present; (2) specificity (true negative rate), which is the probability that a test will be negative when the disease is not present; (3) positive likelihood ratio, which is the ratio between the probability of a positive test result given the presence of the disease and the probability of a positive test result given the absence of the disease. i.e., true positive rate/false positive rate; (4) negative likelihood ratio, which is the ratio between the probability of a negative test result given the presence of the disease and the probability of a negative test result given the absence of the disease. i.e., false negative rate/true negative rate; (5) positive predictive value, which is the probability that the disease is present when the test is positive; (6) negative predictive value, which is the probability that the disease is not present when the test is negative; (7) Accuracy of the test, which is the overall probability that a patient is correctly classified; (8) Prevalence of IR by TG/HDL ratio.

## Results

Table 1 reveals the baseline characteristics of the study population. In total, 815 subjects were selected; among them, 464 (56.93%) were females and 351 (43.07%) were males. The study participants ranged in age from 18 to 60. The mean age for males (44.73 ± 9.87 years) and females (43.12 ± 10.44 years) and the medians of ages were 46 and 44 years, respectively, almost similar across genders, indicating a well-balanced age distribution. The p-value for the difference across genders was 0.2606, which was statistically not significant. The standard deviation and range suggest a broad age representation in both genders.

**Table 1:** Gender-wise characteristics of the study population FBS: fasting blood sugar (mg/dL); FI: fasting insulin (μIU/ml); HOMA-IR: homeostatic model assessment of insulin resistance; TG: triglycerides (mg/dL); HDL: high-density lipoprotein (mg/dL); TG/HDL: triglyceride to high-density lipoprotein ratio; F: female; M: male; Std deviation: standard deviation; min: minimum; max: maximum

Metric	Gender	Mean ±Std deviation	Median	Min - Max	Range	T-test	p-value
Age (years)	M	44.73 ± 9.87	46.00	18.00 - 60.00	42.00	1.2217	0.2606
Age (years)	F	43.12 ± 10.44	44.00	18.00 - 60.00	42.00		
FBS (mg/dL)	M	120.26 ± 56.04	99.00	70.00 - 330.00	260.00	2.4854	0.0131
FBS (mg/dL)	F	111.33 ± 46.15	96.00	70.00 - 326.00	256.00		
FI (μIU/ml)	M	10.32 ± 3.47	9.80	4.50 - 27.40	22.90	1.3323	0.1831
FI (μIU/ml)	F	9.96 ± 3.77	9.00	5.00 - 31.80	26.80		
HOMA-IR	M	3.17 ± 1.95	2.78	0.89 - 9.58	8.69	2.5584	0.0107
HOMA-IR	F	2.83 ± 1.73	2.62	0.97 - 11.56	10.59		
TG (mg/dL)	M	180.01 ± 61.57	184.00	70.00 - 400.00	330.00	2.0408	0.0416
TG (mg/dL)	F	171.37 ± 57.22	178.00	58.00 - 463.00	405.00		
HDL (mg/dL)	M	43.71 ± 6.93	44.00	26.00 - 60.00	34.00	-0.9622	0.3362
HDL (mg/dL)	F	44.16 ± 6.75	44.00	21.00 - 60.00	39.00		
TG/HDL	M	4.25 ± 1.93	3.98	0.08 - 12.92	12.84	2.2751	0.0232
TG/HDL	F	3.96 ± 1.65	3.68	0.08 - 13.62	13.54		

A statistically significant difference (p = 0.0131, significant (S)) was observed in male FBS levels as compared to females, indicating a gender disparity in glucose metabolism. There was a slight difference in FI levels between males and females (p-value = 0.1831, not significant (NS)). The HOMA-IR was higher in males than females, with a statistically significant p-value of 0.0107. Males had higher mean TG levels compared to females (p = 0.0416, S). Females exhibited slightly higher but insignificant HDL levels than males (p = 0.3362, NS). The TG/HDL ratio was significantly higher (p = 0.0232, S) in males than females, suggesting the existence of a less favorable lipid profile and insulin resistance.

Table 2 reflects the differences between anthropometrically obese and non-obese individuals that make them vulnerable to the development of insulin resistance and metabolic syndrome, the ultimate reasons behind cardiovascular diseases. The mean age for obese and non-obese subjects lay around 44.47 ± 10.27 years and 43.46 ± 10.20 years, indicating that obesity development was independent of age and thus could occur at any age. The difference between the ages of obese and non-obese people was statistically not significant (p-value 0.117).

**Table 2:** Obesity status-wise characteristics of the study population FBS: fasting blood sugar (mg/dL); FI: fasting insulin (μIU/ml); HOMA-IR: homeostatic model assessment of insulin resistance; TG: triglycerides (mg/dL); HDL: high-density lipoprotein (mg/dL); TG/HDL: triglyceride to high-density lipoprotein ratio; Std deviation: standard deviation; min: minimum; max: maximum

Metric	Group	Mean ± Std deviation	Median	Min - Max	Range	T-test value	p-value
Age (years)	Obese	44.47 ± 10.27	45.50	19.00 - 60.00	41.00	1.57	0.117
Age (years)	Non-obese	43.46 ± 10.20	45.00	18.00 - 60.00	42.00		
FBS (mg/dL)	Obese	105.98 ± 44.29	95.00	70.00 - 326.00	256.00	9.86	< 0.001
FBS (mg/dL)	Non-obese	99.21 ± 33.06	88.00	65.00 - 195.00	130.00		
FI (μIU/ml)	Obese	10.64 ± 3.63	10.00	5.10 - 27.40	22.30	17.10	< 0.001
FI (μIU/ml)	Non-obese	8.76 ± 2.53	8.40	4.50 - 16.20	11.70		
HOMA-IR	Obese	2.65 ± 1.42	2.50	1.25 - 6.55	5.30	16.01	< 0.001
HOMA-IR	Non-obese	1.87 ± 0.95	1.69	1.12 - 3.89	2.77		
TG (mg/dL)	Obese	178.34 ± 49.22	175.00	111.00 - 305.00	194.00	13.81	< 0.001
TG (mg/dL)	Non-obese	133.21 ± 41.07	129.00	85.00 - 215.00	130.00		
HDL (mg/dL)	Obese	41.63 ± 5.24	44.00	21.00 - 58.00	37.00	-7.41	< 0.001
HDL (mg/dL)	Non-obese	45.22 ± 7.24	44.00	26.00 - 60.00	34.00		
TG/HDL	Obese	5.17 ± 1.69	4.98	2.54 - 13.62	11.08	14.25	< 0.001
TG/HDL	Non-obese	3.50 ± 1.54	3.14	0.08 - 12.92	12.84		

The mean FBS in obese subjects was significantly higher than that in non-obese subjects, indicating glucose metabolism disorders in obese individuals (p-value <0.001, S). Significantly higher mean FI levels in obese than non-obese subjects point towards excessive insulin production due to IR, a hallmark of metabolic syndrome (p-value <0.001, S). Similarly, significantly higher HOMA-IR in obese subjects as compared to non-obese subjects reflected the presence of IR in them (p-value <0.001, S). The mean TG level was much higher in obese subjects than non-obese subjects, with a statistically significant p-value (<0.001), indicating the occurrence of dyslipidemia with hypertriglyceridemia associated with obesity. Obese individuals had lower HDL levels than non-obese individuals, suggesting the presence of low levels of cardioprotective lipoprotein and the presence of a worse cardiovascular risk profile in obesity (p-value <0.001, S). The TG/HDL ratio was statistically significantly higher in obese than non-obese subjects, indicating an adverse lipid profile and severe insulin resistance associated with increased risk for cardiovascular disease in obese individuals (p-value <0.001, S).

Table 3 reveals that there were no significant differences between genders in glycemic status across the different age groups. This lack of significant difference suggests a similar distribution of normal pre-diabetic and diabetic subjects among males and females for each age group, pointing towards uniformity in the selection of subjects and glycemic control or insulin sensitivity across genders.

**Table 3:** Distribution of subjects according to their glycemic status

Age group	Gender	Normal	Pre-diabetic	Diabetic	Total	Chi-square value	p-value
18-30 years	Male	12	9	10	31	0.352	0.553
18-30 years	Female	30	14	12	56		
31-40 years	Male	36	16	18	70	0.005	0.944
31-40 years	Female	67	27	15	109		
41-50 years	Male	70	39	29	138	3.080	0.079
41-50 years	Female	109	36	33	178		
51-60 years	Male	60	22	30	112	1.035	0.309
51-60 years	Female	59	32	30	121		
Total	-	443	195	177	815	-	-

Table 4 demonstrates that no statistically significant gender differences were observed for the age groups 18-30, 41-50, and 51-60 years for diagnosing insulin resistance by HOMA-IR-based criteria, indicating similar levels of insulin resistance between males and females in these age groups (p-value >0.05 for each age group, NS).

**Table 4:** Distribution of insulin-resistant and insulin-sensitive subjects according to HOMA-IR criteria HOMA-IR: homeostatic model assessment of insulin resistance

Age group	Gender	HOMA-IR ≥ 2.5	HOMA-IR < 2.5	Total	Chi-square value	p-value
18-30 years	Male	18	13	31	0.114	0.735
18-30 years	Female	29	27	56		
31-40 years	Male	47	23	70	5.202	0.023
31-40 years	Female	53	56	109		
41-50 years	Male	79	59	138	1.361	0.243
41-50 years	Female	89	89	178		
51-60 years	Male	63	49	112	0.248	0.618
51-60 years	Female	73	48	121		
Total	-	451	364	815	-	-

For the age group of 31-40 years, the male gender influenced IR levels, with potential implications for metabolic health and cardiovascular disease risk (p-value 0.023, S).

Table 5 demonstrates that no statistically significant gender differences were observed for the age groups 18-30, 41-50, and 51-60 years for diagnosing insulin resistance by TG/HDL-based criteria, indicating similar levels of IR between males and females (p-value >0.05 for each age group, NS).

**Table 5:** Distribution of insulin-resistant and insulin-sensitive subjects according to TG/HDL criteria TG/HDL: triglyceride to high-density lipoprotein ratio

Age group	Gender	TG/HDL ≥ 3.5	TG/HDL < 3.5	Total	Chi-square value	p-value
18-30 years	Male	18	13	31	0.0	1.0
18-30 years	Female	33	23	56		
31-40 years	Male	48	22	70	5.545	0.019
31-40 years	Female	54	55	109		
41-50 years	Male	79	59	138	1.140	0.286
41-50 years	Female	90	88	178		
51-60 years	Male	67	45	112	0.041	0.839
51-60 years	Female	75	46	121		
Total	-	464	351	815	-	-

For the age group 31-40 years, male gender influenced IR levels, with potential implications for metabolic health and cardiovascular disease risk, suggesting that this lipid marker may be influenced by gender-specific factors such as hormonal differences, lifestyle, or body fat distribution. (p-value 0.019, S). Insulin resistance assessed by both methods shows similar results.

Table 6 represents that for each age group from 18-30 years to 51-60 years, the T-test value for knowing the difference between IR diagnosed by HOMA-IR and TG/HDL ratio was not statistically significant (p-value >0.05, NS). This indicates that the TG/HDL ratio criteria diagnose IR almost as accurately as the HOMA-IR criteria.

**Table 6:** Age and gender-wise distribution of insulin resistance by different markers HOMA-IR: homeostatic model assessment of insulin resistance; TG/HDL: triglyceride to high-density lipoprotein ratio

Age group	Gender	HOMA-IR ≥ 2.5	TG/HDL ≥ 3.5	T-test value	p-value
18-30 years	Male	18	18	1.286	0.354
18-30 years	Female	29	33		
31-40 years	Male	47	48	1.053	0.293
31-40 years	Female	53	54		
41-50 years	Male	79	79	0.938	0.349
41-50 years	Female	89	90		
51-60 years	Male	63	67	0.798	0.426
51-60 years	Female	73	75		
Total	-	451	464	-	-

Table 7 assesses the efficacy of TG/HDL as a marker of IR as compared to HOMA-IR. We found that TG/HDL had a sensitivity of 91.81% (88.93% to 94.14%; 95% CI) and a specificity of 92.88% (89.67% to 95.34%; 95% CI) for predicting IR. We also found that the test had a positive likelihood ratio of 12.89 (8.83 to 18.83; 95% CI) and a negative likelihood ratio of 0.09 (0.06 to 0.12; 95% CI). The positive predictive value of a test is the probability that the disease is present when the test is positive, which is 94.46% (92.11% to 96.14%; 95% CI), and the negative predictive value of a test is the probability that the disease is not present when the test is negative, which is 89.56% (86.33% to 92.1%; 95% CI). The diagnostic accuracy of the TG/HDL was 92.27% (90.22% to 94.01%; 95% CI), and the disease prevalence according to this marker was 56.93% (53.45% to 60.36%; 95% CI).

**Table 7:** Efficacy of TG/HDL as a marker of insulin resistance as compared to HOMA-IR HOMA-IR: homeostatic model assessment of insulin resistance; TG/HDL: triglyceride to high-density lipoprotein ratio; TP: true positive; FP: false positive; FN: false negative; TN: true negative

Tests	HOMA-IR ≥ 2.5	HOMA-IR < 2.5	Total
TG/HDL ≥ 3.5	426 (TP)	38 (FP)	464
TG/HDL < 3.5	25 (FN)	326 (TN)	351
Total	451	364	815

**Figure 2:**
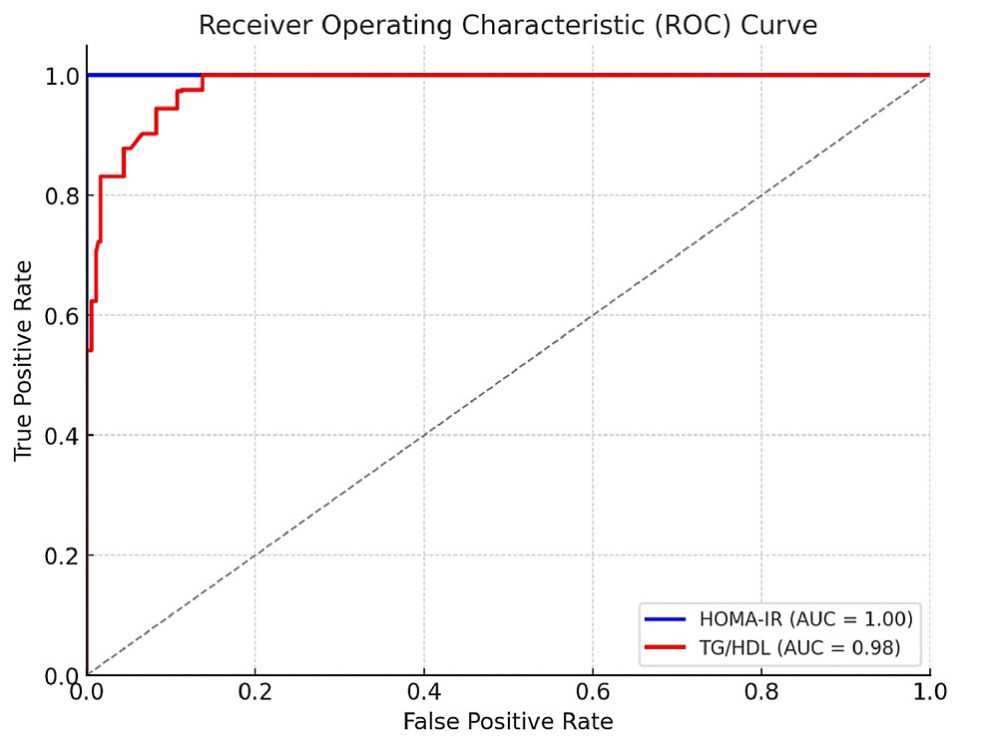
The ROC curve of the TG/HDL ratio against the HOMA-IR value of ≥2.5 ROC curve: receiver operating characteristic curve; AUC: area under the ROC curve; HOMA-IR: homeostatic model assessment for insulin resistance; TG/HDL ratio: triglycerides to high-density lipoprotein ratio

Figure 2 demonstrates the ROC curve of the TG/HDL ratio against a HOMA-IR value of ≥2.5. It shows that, for the study population, the area under the ROC curve (AUC) for HOMA-IR was 1.00, while that for the TG/HDL ratio was 0.98. These results suggest that both markers had excellent discriminative power in identifying IR. The optimal cutoff values for each marker, are determined based on Youden's index, which can be used to classify individuals as insulin-resistant are as follows: optimal cutoff for HOMA-IR: 4.33; optimal cutoff for TG/HDL ratio: 3.45.

Based on the above findings of the ROC curve, for diagnosing IR, the sensitivity of HOMA-IR was 91.81% and that of TG/HDL was 94.46%, while the specificity was 92.88% and 89.56%, respectively. The HOMA-IR and TG/HDL tests had positive predictive values of 94.46% and 91.81% for diagnosing IR, and the negative predictive values of both markers were 89.56% and 92.88%, respectively. These results suggest that the TG/HDL ratio demonstrated a strong correlation with the established IR marker (HOMA-IR) (r = 0.75, p <0.001). A higher TG/HDL ratio was significantly associated with IR in both obese and non-obese subjects in central India (odds ratio (OR) 3.5, 95% CI: 2.4-5.1, p <0.001).

## Discussion

Obesity is a complex multifactorial disease resulting from disproportionate excess body fat, resulting in adverse health outcomes ranging from sleep apnea and dyslipidemias to a range of CVDs [2-4]. Addressing the most basic pathophysiological alteration, i.e., IR, can help in the prevention of the complications of obesity. To diagnose IR, various markers are available, but those are either expensive, complex, invasive, or not suitable for everyday clinical use [9-12], especially in remote and economically disadvantaged areas. As a result, there's a dire need to find a simpler, more cost-effective or affordable, feasible, non-invasive, readily accessible, and trustworthy alternative marker of IR that could be easily used in both medical practice and research contexts. With this idea in mind, the present original study was conducted in adults in rural central India to assess the efficacy of the TG/HDL ratio as a marker of IR by comparing it with a proven marker of IR, HOMA-IR.

To the best of our knowledge, the present study was the first of its kind in central India, as a literature search showed the work done in pediatrics and adolescent age groups in and around India [14, 15]. Central India is known for its demographically unique, diverse, genetically susceptible, and economically poor population.

In total, 815 study participants were selected between the ages of 18 and 60; among them, 464 (56.93%) were females and 351 (43.07%) were males. A study by Steinbeck (2004) [16] demonstrated that the pattern of insulin resistance changes in the adolescent period, so we didn’t include them. Similarly, Refaie et al. (2006) [17] postulated that aging is an inevitable and persistent risk factor for IR. Considering these things, to avoid age-based IR differences in the adult population, we recruited only subjects between the ages of 18 and 60.

Higher values of FBS, FI, HOMA-IR, TG, and TG/HDL in males as compared to females with statistically significant p-values in our study indicate that there exists a gender disparity in glucose metabolism, lipid metabolism, insulin production, beta-cell function, and IR, thus potentially a higher risk of diabetes and related CVD development among males as compared to females.

More or less similar findings were noted by Lin et al. (2022) [18] in their study. When they selected 3,253 men (64.3%) and 1,808 women (35.7%) who had metabolic syndrome (MetS), higher HOMA-IR values or higher IR were found in males, and those males were more prone to CVD and chronic kidney disease. The study conducted by Kohli et al. (2017) [19] in north India among young to middle-aged men (25 years to 44 years) found that lipid abnormalities in terms of deranged TG levels and TG/HDL ratio were found in 51.3% of their study population, and the mean age was 34.7±7.7 years. Kolovou et al. (2009) [20] found that in Greece, females were on average 3.5 years older than males, and they had higher total cholesterol (TC) and HDL levels and low TG and low TC/HDL levels than males. Moreover, males had higher TG and lower HDL levels than females. Higher HDL levels and the protective effect of the estrogen hormone protect women from cardiovascular diseases [21]. These inferences highlight the necessity of considering gender differences in clinical assessments and interventions tailored to address specific health risks associated with metabolic and cardiovascular diseases.

Geer and Shen (2009) [22] found in their study that higher levels of iIR in men compared to women exist due to larger volumes of visceral and hepatic adipose tissue, as well as the absence of any potential protective effects of estrogen. In an article on the epidemiology of type 2 diabetes in India by Pradeepa and Mohan (2021) [23], and another article on socioeconomic inequality in awareness, treatment, and control of diabetes among adults in India, evidence from the National Family Health Survey of India (NFHS), 2019-2021, by Maiti et al. (2023) [24], it was stated that a healthy lifestyle that includes self-maintenance, a balanced diet, and regular physical activity can prevent the onset of IR, irrespective of gender. However, the habits of men and women differ significantly when it comes to putting these preventative guidelines into practice, which is crucial in fighting the metabolic disorder's symptoms. These findings demonstrate that males are more prone to the development of IR, but acquiring a healthy lifestyle can prevent the development of IR in both genders [23, 24].

In the present study, all the metabolic parameters were adversely affected in obese subjects as compared to non-obese subjects, with a statistically significant p-value (<0.001). Obese subjects present with deranged anthropometric indices like BMI, waist circumference (WC), waist-to-hip ratio (WHR), waist-to-height ratio (WHtR), neck circumference (NC), and many more [25-27]. The ill effects of obesity on glucose metabolism and deranged FBS are also reported by Akter et al. (2017) [28]. The findings of these studies are similar to ours. These biochemical and metabolic differences between obese and non-obese individuals make them vulnerable to the development of IR and metabolic syndrome, the ultimate reasons behind CVDs. Lower HDL levels in obese individuals also suggest a worse cardiovascular risk profile and output. Kansal and Kamble (2016) [29] from central India also found that prediabetic people had significantly higher TG/HDL, very low-density lipoprotein (VLDL), TC, LDL, and LDL/HDL ratios when compared to normal, healthy controls, with a statistically significant p-value. Klop et al. (2013) [30] demonstrated in their study similar findings of obesity-related dyslipidemia causing overproduction of TG and VLDL, altered lipid metabolism, and impaired fat processing, resulting in the development of IR and various CVDs. For treatment, they focused on lifestyle changes along with statins and fibrates. They suggested that ApoB and non-HDL-C are more accurate markers for guiding treatment due to their reflection of the atherogenic lipid load. Vittal et al. (2010) [31], Geer et al. (2009) [22], and Misra et al. (2008) [1] demonstrated similar findings in the form of altered glucose and lipid metabolism in obese individuals as compared to non-obese individuals, resulting in raised FBS and altered OGTT.

Wondmkun et al. (2020) [32] described that obesity contributes to diabetes through the release of harmful substances by adipose tissue, which can lead to IR. Factors such as endoplasmic reticulum stress, tissue hypoxia, oxidative stress, and genetic predispositions also play a role. The complex relationship between obesity and type 2 diabetes is largely mediated by IR, a condition influenced by factors like increased fatty acid flux, pro-inflammatory cytokines from adipose tissue, and impaired insulin signaling pathways. Insulin plays a critical role in adipocyte function, promoting triglyceride storage and affecting glucose transport. The mechanisms behind insulin resistance involve altered insulin receptor signaling and glucose transporter expression, with obesity exacerbating these issues through increased fatty acid levels and inflammatory markers. The findings of the abovementioned studies are in accordance with our study findings. Treatments focusing on enhancing insulin sensitivity, such as thiazolidinediones and lifestyle modifications, are key, with ongoing research into the adipose-insulin axis offering potential new therapeutic targets, as stated by Kahn et al. (2000) [33].

In the present study, a significant range in a few markers indicates diversity in metabolic health and the effects of stress, lifestyle, hormones, gender, genetics, and environments on the development or prevention of CVDs. This also suggests the use of multiple variables for assessing metabolic obesity and signifies individualized approaches to health assessments and interventions. The role of an individualized approach, gender differences, and stress levels must be considered for assessing and treating metabolic obesity, even for the same age group of patients. Reed et al. (2017) [34] and Salimzadeh et al. (2016) [35] demonstrated in their study the reason for similar findings: men are more likely to have elevated blood pressure, a higher prevalence of diabetes, and elevated levels of “bad cholesterol” (LDL), outside eating habits; furthermore, the high prevalence of smoking (both active and passive), alcoholism, and genetic and hormonal predisposition make them vulnerable to various cardiovascular diseases.

The stress burden on men outscores everything; resulting in the release of stress hormones that cause a rise in blood pressure, sugar level, and heart rate. [24, 36, 37]. Men neglect their health and symptoms, resulting in delayed presentations at healthcare centers [37]. Estrogen, primarily present in women, has cardioprotective effects, thus making them less vulnerable to CVD in the reproductive age group [21, 36].

A healthy lifestyle, including regular physical activity, a balanced diet, and self-maintenance, can help to prevent the onset of IR, regardless of gender, although the different habits between men and women greatly affect the implementation of preventative guidelines that help in fighting the manifestations of this metabolic disorder [22, 37, 38].

No significant differences were found between genders in glycemic status across the age groups in our study. This lack of significant difference suggests a similar distribution of normal and prediabetic among males and females, pointing towards uniformity in the selection of subjects and glycemic control or insulin sensitivity across genders.

Since HOMA-IR is considered as one of the best standard markers of IR, we considered it an ideal comparison in our study. Singh and Saxena (2010) [39], in their review article, compared various markers of IR and found that HOMA-IR and QUICKI are the best among them; they hadn’t included TG/HDL in their review. Chauhan et al. (2021) [40] also compared TG/HDL with HOMA-IR for assessing IR in prediabetics.

We found that IR by HOMA-IR and by TG/HDL-based criteria reflected that no statistically significant gender differences were observed for the age groups 18-30, 41-50, and 51-60 years (p-value >0.05 for each age group, NS), while males in the age group 31-40 years had more IR than females, indicating potential implications for metabolic health and cardiovascular disease risk (p-value <0.05, S). Klop et al. (2013) [30] demonstrated similar findings of overproduction of TG and VLDL, altered lipid metabolism, and impaired fat processing, resulting in the development of IR and various CVDs. Lin et al. (2022) [18] found in their study that more males had MetS as compared to females, and the higher HOMA-IR values or higher IR in males was a risk factor for CVD and CKD. Reed et al. (2017) [34] and Salimzadeh et al. (2016) [35] demonstrated in their study that men are more likely to have elevated blood pressure, a higher prevalence of diabetes, and elevated levels of “bad cholesterol,” leading to IR and CVD. 

Kim et al. (2011) [41] demonstrated that age, smoking, BMI, alcohol intake, TG level, LDL level, and educational status had a significant impact on HDL values for men, while fat intake was a significant factor for women. After adjusting associated factors, the mean HDL level of 43.8 ± 0.2 mg/dL in men and 46.3 ± 0.2 mg/dL in women, respectively, represents a mean difference of 2.5 mg/dL in Korean adults, which was less than those observed in previous western studies. The findings of Klop et al. (2013) [30], Lin et al. (2022) [18], Salimzadeh et al. (2016) [35], Reed et al. (2017) [34], and Kim et al. (2011) [41] showed a positive correlation with the findings of the present study. Jishna et al. (2023) [42] in their study mentioned the importance of hyperlipidemia control for the treatment of asthma secondary to metabolic syndrome, a consequence of iIR. 

There was no statistically significant difference between IR diagnosed by HOMA-IR and the TG/HDL ratio for each age group in the present study, suggesting that the TG/HDL ratio diagnoses IR almost the same as that of HOMA-IR. Similarly, Flores Guerrero et al. (2024) [43] in their cross-sectional study found that TG/HDL ratio, HOMA-IR, and lipoprotein IR (LP-IR) showed similar associations with carotid intima-media thickness (cIMT) and incident CVD. Behiry et al. (2019) [44] found in their study that TG:HDL ratio ≥1.36, rather than HOMA-IR, is a significant early and sensitive predictor of IR in children.

The findings of Flores Guerrero et al. (2024) [43] and Behiry et al. (2019) [44] were in accordance with the findings of the present study.

As compared to HOMA-IR, the TG/HDL ratio had 91.81% sensitivity and 92.88% specificity, with a positive predictive value of 94.46% and a negative predictive value of 89.56%. The accuracy of the test was 92.27%, and based on these test results, the prevalence of IR by TG/HDL ratio was 56.93%.

Flores Guerrero et al. (2024) [43] in their study found that TG/HDL and HOMA-IR were independently associated with incident CVD, with the TG/HDL ratio showing a slightly stronger association than HOMA-IR, suggesting that lipoprotein-based markers of IR are strongly linked to subclinical and clinical atherosclerosis development, potentially making the measurement of insulin unnecessary for assessing the impact of IR on atherosclerosis. Thus, they proposed that the TG/HDL ratio can serve as a practical alternative marker of IR.

The metabolic syndrome and TG/HDL ratio were found to be positively correlated by Laclaustra et al. (2012) [45], according to the Spanish Metabolic Syndrome in Active Subjects (MESYAS) group, with a prevalence of MetS of 18.8% in men and 6.1% in women. Additionally, as each MetS component is added, the mean TG/HDL value of 2.50 ± 2.2 rises. Furthermore, TG/HDL ratios were two times greater in MetS participants than in non-MetS subjects. With a sensitivity of 80% and a specificity of 78%, they also found that TG/HDL of >2.75 in males and >1.65 in women were highly predictive of MetS.

Kohli et al. (2017) [19] examined the TG/HDL-C ratio pattern and its correlation with other lipid and nonlipid variables in 121 young, middle-aged, and healthy Indian males aged 25-44 and demonstrated a substantial correlation between the TG/HDL-C ratio and other lipid markers as well as adiposity indicators like body fat percentage and BMI in north Indians.

In non-diabetic ACS, the TG/HDL ratio has also been proposed as a predictor of IR in a South Indian study by Rajappa et al. (2014) [46]. According to the study's findings, adult non-diabetic individuals can use the plasma TG/HDL ratio as a marker for various CVD risks in addition to insulin resistance, and it offers a straightforward way to diagnose the condition.

Higher AUCs (1.00 for HOMA-IR and 0.98 for the TG/HDL ratio) suggest the excellent discriminative power of both markers for identifying IR among individuals. The optimal cutoff values of 4.33 for HOMA-IR and 3.45 for TG/HDL predict very high sensitivity and specificity of the HOMA-IR and TG/HDL ratios. The TG/HDL ratio also demonstrated a strong correlation with HOMA-IR (r = 0.75, p <0.001) in both obese and non-obese subjects (OR 3.5, 95% CI: 2.4-5.1, p <0.001). Similar findings were noted by Ghani et al. (2023) [47] in their study of healthy Iraqi adults, in which TG/HDL showed a very good predictive value for IR with an AUC of 0.849, 95% CI = 0.763-0.935, sensitivity = 83%, specificity = 81%, best threshold cut-off value = 3.1, and prevalence of IR = 28.92%.

The gist of all the above-mentioned findings brought us to the conclusion that the TG/HDL ratio is a very good marker of IR in terms of efficacy, simplicity, affordability, non-invasiveness, accessibility, or easy availability tested in a demographically unique, diverse, genetically-susceptible, and economically poor population of central India. Our finding is supported by the findings of Laclaustra et al. (2012) [45], Chauhan et al. (2021) [40], Flores Guerrero et al. (2024) [43], and Behiry et al. (2019) [44], as follows: Laclaustra et al. (2012) [45] concluded in their study that TG/HDL can be regarded as one of the most useful markers in the identification of insulin-resistant states. It also ultimately becomes the risk marker for various adverse outcomes, along with other parameters like plasma TG concentration and insulin concentration. Chauhan et al. (2021) [40] also proposed that the TG/HDL ratio is the easiest to measure of all the indicators of insulin resistance, including HOMA-IR. However, they were unable to find any meaningful link between IR and CIMT. Because CIMT thickening takes a long time to develop, radiological detection of the early stages of the atherosclerotic process has become difficult or hampered. Flores Guerrero et al. (2024) [43] also found that the TG/HDL ratio is equally efficacious as compared to HOMA-IR and can practically be used as a marker of IR and a risk factor for CVD. Behiry et al. (2019) [44] found in their study that TG: HDL ratio ≥1.36, rather than HOMA-IR, is a significant early and sensitive predictor of insulin resistance in children.

Our study, conducted within a hospital setting with a constrained timeframe, presents a few limitations that need acknowledgment. Firstly, the absence of follow-up assessments restricted our ability to observe potential changes occurring over time. Furthermore, the exact causality underlying the observed phenomena has not been determined. External factors beyond our control, such as environmental variables, participant characteristics, or genetic makeup, could have influenced the results. Despite efforts to mitigate confounding variables, the possibility of unidentified factors impacting outcomes remains, underscoring the need for further research to validate and expand upon our findings and conclusion.

## Conclusions

The TG/HDL ratio was an affordable, accessible, and reliable tool for assessing insulin resistance as well as cardiovascular risk in adults. This tool will be of great importance in the early detection and intervention of metabolic obesity and IR. Altered glucose and lipid metabolism and insulin resistance were more common in males and obese subjects. Personalized and gender-based approaches to metabolic health assessment and intervention should be considered for addressing these conditions. Large-scale multicenter studies in the future will be required to validate our findings.

## References

[1] Misra A, Khurana L (2008). Obesity and the metabolic syndrome in developing countries. J Clin Endocrinol Metab.

[2] Lin X, Li H (2021). Obesity: epidemiology, pathophysiology, and therapeutics. Front Endocrinol (Lausanne).

[3] Gupta RD, Tamanna N, Siddika N, Haider SS, Apu EH, Haider MR (2023). Obesity and abdominal obesity in Indian population: findings from a nationally representative study of 698,286 participants. Epidemiologia (Basel).

[4] Shukla A, Kumar K, Singh A (2014). Association between obesity and selected morbidities: a study of BRICS countries. PLoS One.

[5] Reaven GM (1988). Banting lecture 1988. Role of insulin resistance in human disease. Diabetes.

[6] DeFronzo RA, Tobin JD, Andres R (1979). Glucose clamp technique: a method for quantifying insulin secretion and resistance. Am J Physiol.

[7] Mahadik SR, Deo SS, Mehtalia SD (2007). Increased prevalence of metabolic syndrome in non-obese asian Indian-an urban-rural comparison. Metab Syndr Relat Disord.

[8] Mohan V (2004). Why are Indians more prone to diabetes?. J Assoc Physicians India.

[9] Fizelova M, Jauhiainen R, Kangas AJ (2017). Differential associations of inflammatory markers with insulin sensitivity and secretion: the prospective METSIM study. J Clin Endocrinol Metab.

[10] Lau CH, Muniandy S (2011). Novel adiponectin-resistin (AR) and insulin resistance (IRAR) indexes are useful integrated diagnostic biomarkers for insulin resistance, type 2 diabetes and metabolic syndrome: a case control study. Cardiovasc Diabetol.

[11] Jog KS, Eagappan S, Santharam RK, Subbiah S (2023). Comparison of novel biomarkers of insulin resistance with homeostasis model assessment of insulin resistance, its correlation to metabolic syndrome in south Indian population and proposition of population specific cutoffs for these indices. Cureus.

[12] Che B, Zhong C, Zhang R, Pu L, Zhao T, Zhang Y, Han L (2023). Triglyceride-glucose index and triglyceride to high-density lipoprotein cholesterol ratio as potential cardiovascular disease risk factors: an analysis of UK biobank data. Cardiovasc Diabetol.

[13] Cordero A, Ezquerra EA (2009). TG/HDL ratio as surrogate marker for insulin resistance. ESC.

[14] Nur Zati Iwani AK, Jalaludin MY, Yahya A (2022). HDL-C ratio as insulin resistance marker for metabolic syndrome in children with obesity. Front Endocrinol (Lausanne).

[15] Sowndarya K, Joseph J, Shenoy A, Hegde A (2021). Evaluation of triglyceride/high-density lipoprotein ratio as a surrogate marker for insulin resistance in healthy young males. J Nat Sc Biol Med.

[16] Steinbeck KS (2004). Insulin resistance syndrome in children and adolescents: clinical meaning and indication for action. Int J Obes Relat Metab Disord.

[17] Refaie MR, Sayed-Ahmed NA, Bakr AM, Abdel Aziz MY, El Kannishi MH, Abdel-Gawad SS (2006). Aging is an inevitable risk factor for insulin resistance. J Taibah Univ Med Sci.

[18] Lin CA, Li WC, Lin SY (2022). Gender differences in the association between insulin resistance and chronic kidney disease in a Chinese population with metabolic syndrome. Diabetol Metab Syndr.

[19] Kohli A, Siddhu A, Pandey RM, Reddy KS (2017). Relevance of the triglyceride-to-high-density lipoprotein cholesterol ratio as an important lipid fraction in apparently healthy, young, and middle-aged Indian men. Indian J Endocrinol Metab.

[20] Kolovou GD, Anagnostopoulou KK, Damaskos DS (2009). Gender differences in the lipid profile of dyslipidemic subjects. Eur J Intern Med.

[21] Iorga A, Cunningham CM, Moazeni S, Ruffenach G, Umar S, Eghbali M (2017). The protective role of estrogen and estrogen receptors in cardiovascular disease and the controversial use of estrogen therapy. Biol Sex Differ.

[22] Geer EB, Shen W (2009). Gender differences in insulin resistance, body composition, and energy balance. Gend Med.

[23] Pradeepa R, Mohan V (2021). Epidemiology of type 2 diabetes in India. Indian J Ophthalmol.

[24] Maiti S, Akhtar S, Upadhyay AK, Mohanty SK (2023). Socioeconomic inequality in awareness, treatment and control of diabetes among adults in India: Evidence from National Family Health Survey of India (NFHS), 2019-2021. Sci Rep.

[25] Goh LG, Dhaliwal SS, Welborn TA, Lee AH, Della PR (2014). Anthropometric measurements of general and central obesity and the prediction of cardiovascular disease risk in women: a cross-sectional study. BMJ Open.

[26] Mahmoud I, Sulaiman N (2021). Significance and agreement between obesity anthropometric measurements and indices in adults: a population-based study from the United Arab Emirates. BMC Public Health.

[27] Piqueras P, Ballester A, Durá-Gil JV, Martinez-Hervas S, Redón J, Real JT (2021). Anthropometric indicators as a tool for diagnosis of obesity and other health risk factors: a literature review. Front Psychol.

[28] Akter R, Nessa A, Husain MF (2017). Effect of obesity on fasting blood sugar. Mymensingh Med J.

[29] Kansal S, Kamble TK (2016). Lipid profile in prediabetes. J Assoc Physicians India.

[30] Klop B, Elte JW, Cabezas MC (2013). Dyslipidemia in obesity: mechanisms and potential targets. Nutrients.

[31] Vittal BG, Praveen G, Deepak P (2010). Body mass index in healthy individuals and its relationship with fasting blood sugar. J Clin Diagn Res.

[32] Wondmkun YT (2020). Obesity, insulin resistance, and type 2 diabetes: associations and therapeutic implications. Diabetes Metab Syndr Obes.

[33] Kahn BB, Flier JS (2000). Obesity and insulin resistance. J Clin Invest.

[34] Reed RM, Dransfield MT, Eberlein M (2017). Gender differences in first and secondhand smoke exposure, spirometric lung function and cardiometabolic health in the old order Amish: a novel population without female smoking. PLoS One.

[35] Salimzadeh H, Najafipour H, Mirzaiepour F, Navadeh S, Shadkam-Farrokhi M, Mirzazadeh A (2016). Prevalence of active and passive smoking among adult population: findings of a population-based survey in Kerman (KERCADRS), Iran. Addict Health.

[36] Xiang D, Liu Y, Zhou S, Zhou E, Wang Y (2021). Protective effects of estrogen on cardiovascular disease mediated by oxidative stress. Oxid Med Cell Longev.

[37] Bonhomme JJ (2007). Men’s health: impact on women, children and society. J Mens Health Gend.

[38] Ciarambino T, Crispino P, Guarisco G, Giordano M (2023). Gender differences in insulin resistance: new knowledge and perspectives. Curr Issues Mol Biol.

[39] Singh B, Saxena A (2010). Surrogate markers of insulin resistance: a review. World J Diabetes.

[40] Chauhan A, Singhal A, Goyal P (2021). TG/HDL ratio: a marker for insulin resistance and atherosclerosis in prediabetics or not?. J Family Med Prim Care.

[41] Kim HJ, Park HA, Cho YG (2011). Gender difference in the level of HDL cholesterol in Korean adults. Korean J Fam Med.

[42] G J, Abraham E, Verma G (2023). Association of asthma with patients diagnosed with metabolic syndrome: a cohort study in a tertiary care hospital. Cureus.

[43] Flores-Guerrero JL, Been RA, Shalaurova I, Connelly MA, van Dijk PR, Dullaart RP (2024). Triglyceride/HDL cholesterol ratio and lipoprotein insulin resistance score: associations with subclinical atherosclerosis and incident cardiovascular disease. Clin Chim Acta.

[44] Behiry EG, El Nady NM, AbdEl Haie OM, Mattar MK, Magdy A (2019). Evaluation of TG-HDL ratio instead of HOMA ratio as insulin resistance marker in overweight and children with obesity. Endocr Metab Immune Disord Drug Targets.

[45] Laclaustra M, Ordoñez B, Leon M (2012). Metabolic syndrome and coronary heart disease among Spanish male workers: a case-control study of MESYAS. Nutr Metab Cardiovasc Dis.

[46] Rajappa M, Sridhar MG, Balachander J, Sethuraman KR, Rajendiran KS (2014). Lipoprotein ratios as surrogate markers for insulin resistance in south Indians with normoglycemic nondiabetic acute coronary syndrome. ISRN Endocrinol.

[47] Ghani ZA, Qaddori H, Al-Mayah Q (2023). Triglyceride/high-density lipoprotein ratio as a predictor for insulin resistance in a sample of healthy Iraqi adults. J Med Life.

